# Supplementary material for: Uncovering the wider impact of COVID-19 measures on the lives of children with complex care needs and their families: A mixed-methods study protocol
Source: PLoS One. 2024 Jul 8;19(7):e0305543. doi: 10.1371/journal.pone.0305543 (PMC11230525; doi:10.1371/journal.pone.0305543)
Supplement: Attachment — Submitted filename: Response to Reviewers.docx [file pone.0305543.s001.docx]

PLOS ONE

Manuscript ID PONE-D-23-22003

Uncovering the Wider Impact of COVID-19 Measures on the Lives of Children with Complex Care Needs and their Families: a mixed-methods study protocol

Dear editor,

Thank you for providing reviewer feedback. We have included our responses below and have provided the revised manuscript. We believe we have addressed all the comments and that the suggestions have improved the paper.

*1. Please ensure that your manuscript meets PLOS ONE's style requirements, including those for file naming. The PLOS ONE style templates can be found at*

[*https://journals.plos.org/plosone/s/file?id=wjVg/PLOSOne_formatting_sample_main_body.pdf*](https://can01.safelinks.protection.outlook.com/?url=https%3A%2F%2Fjournals.plos.org%2Fplosone%2Fs%2Ffile%3Fid%3DwjVg%2FPLOSOne_formatting_sample_main_body.pdf&data=05%7C01%7Cholly.mcculloch%40iwk.nshealth.ca%7C03dbc0c5ef274900f0e208dbdc758f8b%7C8eb23313ce754345a56a297a2412b4db%7C0%7C0%7C638346169851098784%7CUnknown%7CTWFpbGZsb3d8eyJWIjoiMC4wLjAwMDAiLCJQIjoiV2luMzIiLCJBTiI6Ik1haWwiLCJXVCI6Mn0%3D%7C3000%7C%7C%7C&sdata=GFE8kf5ENeJcZCRLXNtDwEYMaAiG58sUYpmZWR%2BvG2c%3D&reserved=0) *and*

[*https://journals.plos.org/plosone/s/file?id=ba62/PLOSOne_formatting_sample_title_authors_affiliations.pdf*](https://can01.safelinks.protection.outlook.com/?url=https%3A%2F%2Fjournals.plos.org%2Fplosone%2Fs%2Ffile%3Fid%3Dba62%2FPLOSOne_formatting_sample_title_authors_affiliations.pdf&data=05%7C01%7Cholly.mcculloch%40iwk.nshealth.ca%7C03dbc0c5ef274900f0e208dbdc758f8b%7C8eb23313ce754345a56a297a2412b4db%7C0%7C0%7C638346169851098784%7CUnknown%7CTWFpbGZsb3d8eyJWIjoiMC4wLjAwMDAiLCJQIjoiV2luMzIiLCJBTiI6Ik1haWwiLCJXVCI6Mn0%3D%7C3000%7C%7C%7C&sdata=eteM8d4rspG1ne8yJUsqD1MXA4hnZYqmEYdg%2FesisUE%3D&reserved=0)

Thank you. All the files are in accordance with the required naming convention and the manuscript meets the style requirements.

*2. Thank you for stating the following financial disclosure:*

*“This work is supported by Canadian Institutes of Health Research, Operating Grant: Addressing the Wider Health Impacts of COVID-19. Grant #473640 Dr. Janet Curran is the Nominated Principal Applicant”*

*Please state what role the funders took in the study.  If the funders had no role, please state: "**The funders had no role in study design, data collection and analysis, decision to publish, or preparation of the manuscript."*

*If this statement is not correct you must amend it as needed.*

*Please include this amended Role of Funder statement in your cover letter; we will change the online submission form on your behalf.*

Thank you. We have amended the financial disclosure as advised in both the manuscript and the cover letter.

*3.* *We note that you have referenced (Lane J. Operationalizing Intersectionality Theory in Research. Dalhous Univ. in preparation) which has currently not yet been accepted for publication. Please remove this from your References and amend this to state in the body of your manuscript: (ie “Bewick et al. [Unpublished]”) as detailed online in our guide for authors*

[*http://journals.plos.org/plosone/s/submission-guidelines#loc-reference-style*](https://can01.safelinks.protection.outlook.com/?url=http%3A%2F%2Fjournals.plos.org%2Fplosone%2Fs%2Fsubmission-guidelines%23loc-reference-style&data=05%7C01%7Cholly.mcculloch%40iwk.nshealth.ca%7C03dbc0c5ef274900f0e208dbdc758f8b%7C8eb23313ce754345a56a297a2412b4db%7C0%7C0%7C638346169851098784%7CUnknown%7CTWFpbGZsb3d8eyJWIjoiMC4wLjAwMDAiLCJQIjoiV2luMzIiLCJBTiI6Ik1haWwiLCJXVCI6Mn0%3D%7C3000%7C%7C%7C&sdata=Tyl5GAsSjfDl3r8i9TsuLn93dvr6eSpTo%2F56oFVoyg8%3D&reserved=0)

Thank you. We have removed all references that have not yet been accepted for publication.

*4. Your ethics statement should only appear in the Methods section of your manuscript. If your ethics statement is written in any section besides the Methods, please delete it from any other section.*

Thank you. We have removed the ethics statement from all sections besides the Methods.

*5. Please review your reference list to ensure that it is complete and correct. If you have cited papers that have been retracted, please include the rationale for doing so in the manuscript text, or remove these references and replace them with relevant current references. Any changes to the reference list should be mentioned in the rebuttal letter that accompanies your revised manuscript. If you need to cite a retracted article, indicate the article’s retracted status in the References list and also include a citation and full reference for the retraction notice.*

Thank you. A full review of the reference list was performed, and it is complete and correct. Reference 26 was updated from the accepted to the published citation.

**Comments to the Author**

*1. Does the manuscript provide a valid rationale for the proposed study, with clearly identified and justified research questions?

The research question outlined is expected to address a valid academic problem or topic and contribute to the base of knowledge in the field.*

*Reviewer #1: Yes*

Thank you.

*2. Is the protocol technically sound and planned in a manner that will lead to a meaningful outcome and allow testing the stated hypotheses?

The manuscript should describe the methods in sufficient detail to prevent undisclosed flexibility in the experimental procedure or analysis pipeline, including sufficient outcome-neutral conditions (e.g. necessary controls, absence of floor or ceiling effects) to test the proposed hypotheses and a statistical power analysis where applicable. As there may be aspects of the methodology and analysis which can only be refined once the work is undertaken, authors should outline potential assumptions and explicitly describe what aspects of the proposed analyses, if any, are exploratory.*

*Reviewer #1: Partly*

Thank you. We have added further clarity to the definition of complex care that we are using for this project to increase the clarity.

*3. Is the methodology feasible and described in sufficient detail to allow the work to be replicable?


Descriptions of methods and materials in the protocol should be reported in sufficient detail for another researcher to reproduce all experiments and analyses. The protocol should describe the appropriate controls, sample size calculations, and replication needed to ensure that the data are robust and reproducible.*

*Reviewer #1: Yes*

Thank you.

*4. Have the authors described where all data underlying the findings will be made available when the study is complete?

The* [*PLOS Data policy*](https://can01.safelinks.protection.outlook.com/?url=https%3A%2F%2Fjournals.plos.org%2Fplosone%2Fs%2Fmaterials-and-software-sharing&data=05%7C01%7Cholly.mcculloch%40iwk.nshealth.ca%7C03dbc0c5ef274900f0e208dbdc758f8b%7C8eb23313ce754345a56a297a2412b4db%7C0%7C0%7C638346169851098784%7CUnknown%7CTWFpbGZsb3d8eyJWIjoiMC4wLjAwMDAiLCJQIjoiV2luMzIiLCJBTiI6Ik1haWwiLCJXVCI6Mn0%3D%7C3000%7C%7C%7C&sdata=MSkwHMbc603x%2FUXvWCzZUcd5Cr%2F1Mg9Eh5Rss48lkn0%3D&reserved=0) *requires authors to make all data underlying the findings described in their manuscript fully available without restriction, with rare exception, at the time of publication. The data should be provided as part of the manuscript or its supporting information, or deposited to a public repository. For example, in addition to summary statistics, the data points behind means, medians and variance measures should be available. If there are restrictions on publicly sharing data—e.g. participant privacy or use of data from a third party—those must be specified.*

*Reviewer #1: Yes*

Thank you.

*5. Is the manuscript presented in an intelligible fashion and written in standard English?

PLOS ONE does not copyedit accepted manuscripts, so the language in submitted articles must be clear, correct, and unambiguous. Any typographical or grammatical errors should be corrected at revision, so please note any specific errors here.*

*Reviewer #1: Yes*

Thank you.

*6. Review Comments to the Author

Please use the space provided to explain your answers to the questions above and, if applicable, provide comments about issues authors must address before this protocol can be accepted for publication. You may also include additional comments for the author, including concerns about research or publication ethics.

You may also provide optional suggestions and comments to authors that they might find helpful in planning their study.


(Please upload your review as an attachment if it exceeds 20,000 characters)*

*Reviewer #1: This is an interesting protocol to research the effects of the COVID 19 measures on the care of children with complex needs.

It is not clear however, what is meant by complex needs and from the way the protocol is presented there is no specific gradient regarding the severity of each case. From what it is written, the interview in Phase 2 will be with the child, so cases of complex needs that impede communication are hereby excluded.*

Thank you. As we state in lines 74-77, complex care needs for this project are defined as multidimensional health and social care needs. The Phase 2 interviews will be conducted with youth and/or caregivers. Therefore, should a child have a case of complex needs that impedes communication, their caregiver will be interviewed.

*Recall bias is an important limitation. Perhaps it would be helpful to carefully map the current state of care of these children (post pandemic) with information collected during the interview as well as from the services (eligibility for benefits, visits to the IWK health care unit etc) to facilitate comparison with the pandemic period.*

Thank you. We agree that recall bias is an important limitation to address in this work. This project is looking specifically at the impact during the pandemic, so we do not intend to compare to the post-pandemic period. We have included the environmental scan to have a point of reference to remind both key informants and families of the key changes during the pandemic to clarify their impact.
